# Studies on macrofungi diversity and discovery of new species of *Abortiporus* from Baotianman World Biosphere Reserve

**DOI:** 10.1515/biol-2022-0614

**Published:** 2023-05-27

**Authors:** Lu Tie, Zhao Lang, Li Deng, Zhao Junqiang

**Affiliations:** College of Life Science and Engineering, Henan University of Urban Construction, Pingdingshan, 467036, PR China; Zhengzhou Foreign Language School, Zhengzhou, 452470, PR China; Xuchang Customs of the People’s Republic of China, 461000 Xuchang, Henan, China

**Keywords:** Baotianman Biosphere Reserve, macrofungi, species diversity, *Abortiporus*

## Abstract

This research focuses on macrofungi in Baotianman Biosphere Reserve and their relationships with plant ecosystems. The findings demonstrate the reserve’s macrofungal resources. The study collected 832 specimens, among which 351 macrofungi species were identified, belonging to six classes, 19 orders, 54 familiae, and 124 genera, and one new species of *Abortiporus* was found. Among them, 11 familiae with a total of 231 species were dominated, accounting for 20.37% of the total number of familiae and 65.81% of the total number of species; 14 genera with a total of 147 species were dominated, accounting for about 11.38% the total number of genera and 41.88% of the total number of species. The richness of macrofungi at the species level was considerably different across the four vegetation types in the reserve, showing that the vegetation types had a bigger influence on macrofungi. In the evaluation of macrofungal resources, a total of 196 species of edible fungi, 121 species of medicinal fungi, 52 species of poisonous fungi, and 37 species of macrofungi with unclear economic value were counted. *Abortiporus baotianmanensis* is a new species of podoscyphaceae in the genus *Abortiporus*. The new species display the reserve’s richness. Next, the project seeks to generate and conserve macrofungal resources.

## Graphical abstract

**Figure:**
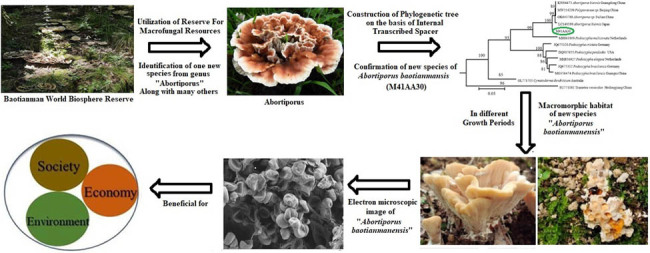

## 1 Introduction

Macrofungi are fungi that can form visible fruiting bodies, most of which belong to the *Basidiomycota* and a few to the *Ascomycota*. As a crucial coenotype in the fungi, macrofungi are vital in stabilizing the ecological balance, having a high application value. Biodiversity plays a decisive role in maintaining the balance of the ecosystem. As an essential part of the ecosystem, macrofungi not only play an important role in maintaining the ecological balance but also are becoming an indispensable part of human production and life [1,2,3]. According to research findings, there are a total of about 1.5 million species of fungi in the world, of which about 70,000 species have been described, including about 14,000 macrofungi species. There are 9,302 species recorded in China, including 1,789 edible fungi, 798 medicinal fungi, and 561 edible and medicinal fungi [4,5]. Macrofungi are closely related to human life. At present, edible fungi have played an increasingly important role in people’s lives; however, due to insufficient understanding, accidental ingestion of toxic fungi frequently occurs. Therefore, studying macrofungi diversity is an arduous and urgent task, and it is the most effective way to grasp the status of species diversity, including the threatened status and degree.

The Baotianman Biosphere Reserve is located at the southern foot of Funiu Mountain, north of Xiaguan Town, Neixiang County, Nanyang City, Henan Province, China. It is the transition zone from the warm temperate zone to the north subtropical zone and from the second step to the third step and a well-preserved comprehensive gene bank in central China. The geographical coordinates of the area are 33°20′∼33°36′N, 111°47′∼112°04′E, with the highest altitude of 1,830 m and an average altitude of 1,400 m. The average annual temperature is 15.1°C. The annual average evaporation is 991.6 mm, the annual average precipitation is 855.6 mm, and the annual average relative humidity is 68% [6]. It belongs to the north subtropical mixed evergreen deciduous broad-leaved forest belt, with rich plant and animal species [7]. In 2009, Haiyou et al. carried out a preliminary investigation on the macrofungal resources in the Funiu Mountain area, and there were 166 species of macrofungi in 11 familiae [8]. Juan et al. investigated the Polyporaceaes in the Baotianman Biosphere Reserve and identified 106 Polyporaceae in 50 genera [9]. In 2017, Du et al. and Lixian et al. investigated the diversity of macrofungi species in Funiu Mountain [10,11]. According to numerous studies, the majority of current research on the Baotianman Biosphere Reserve focuses on animals, plants, and microorganisms. For instance, Zuomin and Naiqun [12,13] studied the diversity of plant communities in the Baotianman Biosphere Reserve, and Gao [14] studied the diversity of slime molds in the Baotianman Biosphere Reserve. However, restricted by various factors, the species of fungi are still unclear in this area, and further investigation and analysis are needed. The habitat and utilization value of certain fungi also need to be further studied. There is an urgent need to supplement the data on species diversity and floristic distribution of macrofungi in this reserve, to provide a scientific basis for the rational utilization and protection of macrofungal resources.

## 2 Materials and methods

### 2.1 Research materials

The macrofungi specimens for this experiment were collected in the Baotianman Biosphere Reserve from July to October 2020 and May to October 2021. More than 800 macrofungi fruiting body specimens were used for the experiment and stored in the specimen library at Henan Urban Construction College.

### 2.2 Specimen identification

The specimens were collected with the sampling method and random inspection method. Based on the different occurrence times of macrofungi fruiting bodies and the seasonal changes in environmental factors such as temperature, air humidity, and lighting, the specimens were collected 1–2 times per month at the appropriate time from May to October. The macrofungi collected grew on various substrates, such as fallen trees, tree trunks, litter, and ground surfaces. The morphological characteristics of the mature fruiting bodies were observed and recorded in detail with wildlife photographs. The identification method was based on traditional taxonomy and molecular biology method. The quantitative features were incorporated from 2–5 fruiting bodies. The spore size was defined by the average data of 20 spores. Traditional taxonomy included morphological and microscopic identification, which featured a general identification practice conducted by examining materials such as monographs, references, and color illustrations while referring to field records and photos. Microscopic observation was carried out by immersing the materials in distilled water, 1% KOH solution, and 1% Congo red stain. Finally, molecular biology techniques were applied to identify specimens that did not apply to traditional taxonomy. First, DNA was extracted with the improved cetyltrimethyl ammonium bromide method, and the ITS sequence was amplified by PCR. The obtained sequence results were analyzed and compared in Genbank by BLAST to check the homology between the researched fungi and the target species included in Genbank.

### 2.3 Quadrat setting

The Baotianman Biosphere Reserve is located at the north–south boundary of vegetation in China and is the transition zone from warm temperate deciduous broadleaf forests to subtropical evergreen broadleaf forests, with rich vegetation types. Under the influence of climate, the vegetation distribution changes obviously with altitude. Based on the research of Shi [15] on the vegetation community in the Baotianman area and combined with the selected sample plots, the vegetation in the Baotianman area can be roughly divided into broadleaf forests, coniferous forests, coniferous-broadleaf mixed forests, and shrubs. The study selects 10 quadrats with a size of 10 m × 10 m for each community and counts the species composition and quantity of species of macrofungi.

### 2.4 Diversity index analysis

For the fungal specimens collected in the Baotianman Biosphere Reserve, a calculation was performed to define the dominant familiae and genera. The α diversity index was selected to analyze the diversity of macrofungi collected in different communities and to explore the influence of different vegetation types on the distribution of macrofungi.

Based on the obtained dictionary of macrofungi in the Baotianman Biosphere Reserve, floristic composition analysis was carried out to analyze the statistics of familiae, genera, and species. Based on the number of species and descending order, the familiae with more than ten species were identified as the dominant one, whereas the genus with more than five species was identified as the dominant one in descending order.

The diversity of the floristic community was obtained from the study by Keping and Liu [16]. Simpson’s diversity index *D*, Shannon–Weaver’s index *H*, Menhinick’s abundance index *R*, and Pielou’s evenness index *E* were used as follows:

(1) D=1-\sum {P}_{i}^{2},]

(2) H=-\sum {P}_{i}\hspace{.25em}\text{ln}\hspace{.25em}{P}_{i},]

(3) R=S/\sqrt{N},]

(4) E=H/\text{ln}\hspace{.25em}S,]

where *P*_*i*_ means the proportion of the number of individuals of the *i*th species to the total number of individuals of all species in the vegetation area; *S* means the total number of species in the vegetation area; *N* means the total number of individuals in the vegetation area, *i* = 1, 2, 3,… *S*.

### 2.5 Resource evaluation method

The species diversity dictionary of the identified specimens was analyzed and filed according to the 10th edition of the *Dictionary of The Fungi* and the Fungi Indexed Database. The edible fungi, medicinal fungi, and toxic fungi were specifically classified with the methods of Zhou and Yang [17], and Dai and Yang [18] and Bau et al. [19].

## 3 Results and analysis

The Baotianman World Biosphere Reserve is rich in macrofungi. After the process, 351 species of macrofungi were identified in the Baotianman Biosphere Reserve (Tables 1 and 2), belonging to six classes, 19 orders, 54 familiae, and 124 genera, including two classes, 12 orders, 45 familiae, and 115 genera, of *Basidiomycota* and four classes, seven orders, nine familiae and nine genera of *Ascomycota*, and one new species of *Abortiporus*.

**Table 1:** Statistical table of macrofungi in Baotianman National Nature Reserve

Division	Classis	Order	Familia	Genus	Species
*Ascomycota*	4	7	9	9	13
*Basidiomycota*	2	12	45	115	338
Total	6	19	54	124	351

**Table 2:** Number of families, genera, and species of macrofungi in Baotianman Mountain National Nature Reserve

Familia	No. of genera	No. of species	Family	No. of genera	No. of species
Leotiaceae	1	1	Mycenaceae	3	4
Sclerotiniaceae	1	1	Pleurotaceae	2	8
Phacidiaceae	1	1	**Lycoperdaceae**	**3**	**16**
Helvellaceae	1	2	**Amanitaceae**	**1**	**23**
Morohellaceae	1	2	Sclerodermataceae	2	4
Pyronemataceae	1	1	**Boletaceae**	**6**	**19**
Hypoxylaceae	1	1	Gomphidiaceae	1	1
Cordycipitaceae	1	3	Paxillaceae	1	2
Geoglossaceae	1	1	Suillaceae	2	4
Tremellaceae	1	4	Gyroporaceae	1	1
**Hymenochaetaceae**	**3**	**10**	Hydnaceae	2	4
Bankeraceae	1	1	Geastraceae	1	2
Thelephoraceae	1	1	Bondarzewiaceae	1	1
Auriculariaceae	2	4	Steccherinaceae	1	1
**Tricholomataceae**	**11**	**25**	Meruliaceae	4	4
**Agaricaceae**	**3**	**16**	Panaceae	1	2
Psathyrellaceae	2	9	**Polyporaceae**	**15**	**40**
Cortinariaceae	3	8	Incrustoporiaceae	1	2
Lyophyllaceae	1	1	Phanerochaetaceae	2	3
Inocybaceae	2	6	Entolomataceae	2	4
Hygrophoraceae	1	4	Auriscalpiaceae	1	1
**Pluteaceae**	**5**	**16**	**Russulaceae**	**2**	**41**
**Strophariaceae**	**4**	**11**	Stereaceae	1	2
**Clavariaceae**	**5**	**14**	Phallaceae	6	9
Schizophyllaceae	1	1	Dacrymycetaceae	1	2
Bolbitiaceae	1	1	Incertae sedis	5	5

Note: Bold font indicates the dominant familiae.

### 3.1 Statistics and analysis of dominant familiae and genera

Based on the analysis of the sorted familiae, genera, and species, there are 11 familiae with more than (or equal to) 10 species (Table 3), accounting for 20.37% of the total number of familiae, and a total of 231 species, accounting for 65.81% of the total species. The family with the most species is Russulaceae with 41 species, accounting for 11.68% of the total species, followed by Polyporaceae with 40 species, accounting for 11.40% of the total species. However, the previous studies also reported the isolation of 106 species from the Polyporaceae Baotianman Biosphere Reserve in 2009 and 124 species in 2015 [9,20]. Similarly, the Baotianman Biosphere Reserve has also reported the isolation of Russulaceae [21]. The rest are Tricholomataceae with 25 species, Amanitaceae with 23 species, Boletaceae with 19 species, Pluteaceae, Lycoperdaceae, and Agaricaceae with 16 species, Clavariaceae with 14 species, Strophariaceae with 11 species and Hymenochaetaceae with 10 species, accounting for 7.12, 6.55, 5.41, 4.56, 4.56, 4.56, 3.99, 3.13, and 2.85% of the total species, respectively. There are 40 familiae with less than 10 species, accounting for about 79.63% of the total familiae, and a total of 16 species, accounting for about 32.48% of the total species, belonging to the affiliation in this flora.

**Table 3:** The survey of advantage families (≥10 species) of Macrofungi in Baotianman National Nature Reserve

Familia	No. of species	Ratio of species number to the total (%)
Russulaceae	41	11.68
Polyporaceae	40	11.40
Tricholomataceae	25	7.12
Amanitaceae	23	6.55
Boletaceae	19	5.41
Pluteaceae	16	4.56
Lycoperdaceae	16	4.56
Agaricaceae	16	4.56
Clavariaceae	14	3.99
Strophariaceae	11	3.13
Hymenochaetaceae	10	2.85
Total	231	65.81

Based on the statistics, there are 123 genera and 351 species of macrofungi in Baotianman Biosphere Reserve, including 14 genera with more than five species (or equal to five species [Table 4]), and a total of 147 species, accounting for 11.38% of the total genera and 41.88% of the total number of species in the reserve. Among them, *Russula* has the most species, which are 29 in total, accounting for 8.26% of the total species, followed by *Amanita* with 23 species, accounting for 6.55% of the total species. The rest are *Agaricus* with 13 species, *Lactarius* with 12 species and *Lycoperdon* with 11 species, accounting for 3.70, 3.42, and 3.13% of the total species, respectively; *Boletus* and *Trametes* with eight species, *Phellinus* and *Marasmius* with seven species, *Coprinopsis*, *Lepiota*, *Ramaria* and *Pleurotus* with six species, and *Coriolus* with five species, accounting for 2.28, 1.99, 1.70, and 1.42% of the total species, respectively. There are 109 genera with less than five species, accounting for 88.62% of the total genera and a total of 204 species, accounting for 58.12% of the total species.

**Table 4:** The survey of advantage genera (≥5 species) of Macrofungi in Baotianman National Nature Reserve

Genus	No. of species	Ratio of species number to the total (%)
*Russula*	29	8.26
*Amanita*	23	6.55
*Agaricus*	13	3.70
*Lactarius*	12	3.42
*Lycoperdon*	11	3.13
*Boletus*	8	2.28
*Trametes*	8	2.28
*Phellinus*	7	1.99
*Marasmius*	7	1.99
*Coprinopsis*	6	1.70
*Lepiota*	6	1.70
*Ramaria*	6	1.70
*Pleurotus*	6	1.70
*Coriolus*	5	1.42
Total	147	41.88

### 3.2 Diversity analysis of macrofungi in different types of vegetation

The distribution of macrofungi in four different types of vegetation was obtained by the statistics, and the data analysis was carried out for Simpson’s diversity index *D*, Shannon–Weaver’s index *H*, Menhinick’s richness index *R* and Pielou’s evenness index *E* in different vegetation communities in Baotianman Biosphere Reserve (Table 5).

**Table 5:** The macrofungi diversity index in different vegetation types in the Baotianman National Nature Reserve

Vegetation community types	Species	*N*	Diversity index		Richness index *R*	Evenness index
			*D*	*H*		
Type Ⅰ	404	289	0.9958	5.7749	14.3783	1.0191
Type Ⅱ	158	119	0.9902	3.7050	9.4671	0.7752
Type Ⅲ	231	172	0.9931	5.0890	11.3168	0.9886
Type Ⅳ	39	36	0.9691	3.5435	5.7646	0.9888

The results show that, for the four different types of vegetation in Baotianman Biosphere Reserve, the macrofungal species richness *R* was Community I > Community III > Community II > Community IV, with large differences. This indicates that the composition of the vegetation has a significant impact on the distribution of macrofungi.

Community I has the highest species richness of 14.3783, and 289 species were identified in the 404 specimens collected. Among others, *Russula*, *Agaricus*, *Marasmius*, *Lactarius,* and *Boletus*, which are dominant, total of 32 species, accounting for 31.83% of the total species of this community. Community I is located in the reserve’s core area, where plant structure is mainly dominated by *Quercus aliena*, and many macrofungi are symbiotic with Quercus and understory shrubs. A previous investigation also reported *Quercus aliena* from the same area in April [22]. Another report also suggested the existence of *Quercus* species in the area in recent years [23], indicating that the community structure was stable and naturally regenerated well [24]. This area has high canopy density, high relative humidity, strong water storage capacity, thick dead branches and leaves, and many fallen rot trees in the forest. More humus can form under such circumstances, providing a favorable growth environment for macrofungi.

The following is Community III, with a richness index of 11.3168, and 172 species were identified in the 231 specimens collected, including 66 species of *Amaita*, *Phellinus*, *Coriolus,* and *Trametes*, accounting for 38.37% of the total species in this community. The vegetation in the community is dominated by *Quercus aliena* and *Pinus armandii*. A previous investigation demonstrated the domination of *Pinus armandii* species in the same area [25]. The community structure was a mixed coniferous and broad-leaf forest, with understory overgrown weeds. There are certain difficulties in the collection process, and omissions are inevitable. Therefore, this area has a low richness index. The species richness of Community II was 9.4671, and 119 species were identified in the 158 specimens collected. Among others, *Amaita*, *Coprinus*, *Pleurotus,* and *Ramaria* are dominant, a total of 44 species, accounting for 36.97% of the total species in this community. The community is dominated by *Pnus armandii* and supplemented by some Fagaceae plants. Due to the high altitude and low canopy density of the coniferous forest, even under high relative humidity, the low temperature at high altitude limits the growth of macrofungi, so the richness index is low.

At the same time, although the richness index of Community I is greater than that of Communities II and III, the proportion of dominant genera in Communities II and III is larger than that in Community I. In this case, it is found that Amanita is dominated in coniferous forests and mixed coniferous and broadleaf forests. Based on the data, most *Amanita* can form ectomycorrhizal fungi with Acicularidae or Fagaceae. Their growth conditions are harsh, and there is no artificial cultivation [26]. It shows that there are more diverse tree species in the reserve, with rich ecological diversity.

The minimum species richness of Community IV is 5.7646, and 36 species were identified in the 39 specimens collected. Among others, *Agaricus* is dominant, with a total of five species, accounting for 13.89% of the total species in this community. Community IV is mainly dominated by Cornaceae and Oleaceae, including *Cornus kousa*, *Forsythia suspensa,* and other plants, which compete fiercely with macrofungi; therefore, both the occurrence probability of macrofungi and the species richness index decreased.

**Figure 1:**
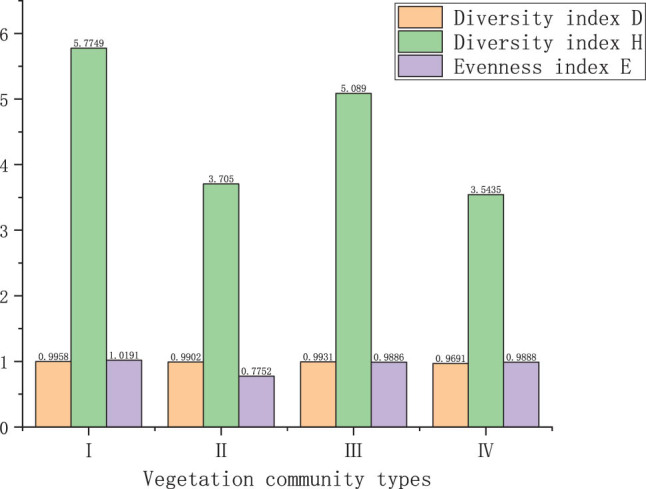
The macrofungi diversity index and species evenness index in different vegetation type.

The diversity indexes *D* and *H* are Community I > Community III > Community II > Community IV. With the two measurement methods, the variation trend is consistent (Figure 1), and the understory vegetation types of Community I and Community III are rich, with fast natural regeneration, which is conducive to the occurrence of macrofungi. Located at a higher altitude, Community II has high relative humidity and low temperature, which is not conducive to the occurrence of macrofungi. The main vegetation of Community IV is dominated by Cornaceae, with a single vegetation type, slow natural regeneration, low canopy density, and long lighting time, limiting the growth of macrofungi.

Judging from the evenness index *E*, the evenness indexes of Communities I, III, and IV have no significant difference; while Community III is a mixed coniferous and broadleaf forest, with complex vegetation types and dense understory vegetation, so sampling might be missed and the evenness index is lower than that of Community I. Community IV is dominated by shrubs, which compete fiercely with the growth of macrofungi, resulting in fewer types and numbers of macrofungi, so the evenness is high. The minimum evenness index of Community II is 0.7752. Because Community II is a coniferous forest with a large number of Amanita, the evenness index of this community is the lowest. It is speculated that there may be tree species symbiotic with amanita in Community II, or the environment may be conducive to the growth of Amanita.

### 3.3 Evaluation of macrofungal resources

Baotianman Biosphere Reserve is rich in macrofungal resources. The evaluation of macrofungal resources in the reserve was carried out to evaluate the resource value and to provide a theoretical basis for the development and utilization of macrofungi. This article referred to the classification method for the macrofungi in Wuling Mountain developed by Yang et al. [27] From the perspective of economic value, the wild macrofungi with economic attributes in this reserve were sorted and classified into four categories as follows: edible fungi, medicinal fungi, poisonous fungi, and fungi with unknown application value. Among them, there are 196 species of edible fungi, accounting for 55.84% of the total; 121 species of medicinal fungi, accounting for 34.47% of the total; 52 species of poisonous fungi, accounting for 14.81% of the total, and 37 species of macrofungi with unknown application value. According to the statistics, there is a large proportion of macrofungi with economic value, indicating that the reserve has great development and utilization value.

### 3.4 Edible fungi

Among the identified 351 species of Baotianman macrofungi, 196 species are classified as edible, among which 17 species can be cultivated, including *Agaricus arvensis* sensu Cooke, *Agaricus bitorquis* (Quél.) Sacc., *Agaricus campestris* sensu Cooke, *Lyophyllum cinerescens* (Bull.) Konrad, *Volvariella volvacea* (Bull.) Singer, *Pholiota aurivella* (Batsch) P. Kumm., *Pholiota adiposa* (Batsch) P. Kumm., *Agrocybepraecox* (Pers.) Fayod, *Fistulina hepatica* (Schaeff.) With., *Schizophyllum commune* Fr., *Pleurotus cornucopiae* (Paulet) Quél., *Pleurotus ostreatus* sensu Cooke, *Pleurotus pulmonarius* sensu auct., *Pleurotus sapidus* Quél., *Pleurotus citrinopileatus* Singer, *Hohenbuehelia serotina* (Pers.) Singer, and *Dictyophora indusiata* sensu auct. brit.

### 3.5 Medicinal fungi

One hundred and twenty-one medicinal fungi are found, among which 11 species can be cultivated as *Morchella crassipes* (Vent.) Pers., *Cordyceps militaris* (L.) Fr., *Tremella fuciformis* Berk., *Tremella lutescens* Pers., *Auricularia auricula* (L.) Underw., *Agaricus arvensis* sensu Cooke, *Agaricus campestris* sensu Cooke, *Lentinus edodes* (Berk.) Singer, *Ganoderma lucidum* (Curtis) P. Karst., *Wolfiporia cocos* (F.A. Wolf) Ryvarden, and *Dictyophora indusiata* sensu auct. brit.

### 3.6 Poisonous fungi

According to the statistics, there are a total of 52 species of poisonous fungi in the Bantianman Biosphere Reserve, and the poisoning types of poisonous fungi are mainly Gastroenteritis, Psychoneurological disorder, Hepatic damage type, Hemolytic type, Multi-organ damage type, Respiratory and circulatory failure type, etc. Among them, the *Amanita* has a large number of species, with 14 species, accounting for 29.17% of the total poisonous fungi. The possible reason lies in that the reserve has a certain number of fagaceae and acicular vegetation that forms the ectomycorrhizal fungi with most amanita, indicating that the reserve has an excellent ecological environment and development and utilization value.

### 3.7 Identification of new species of *Abortiporus*

**Figure 2:**
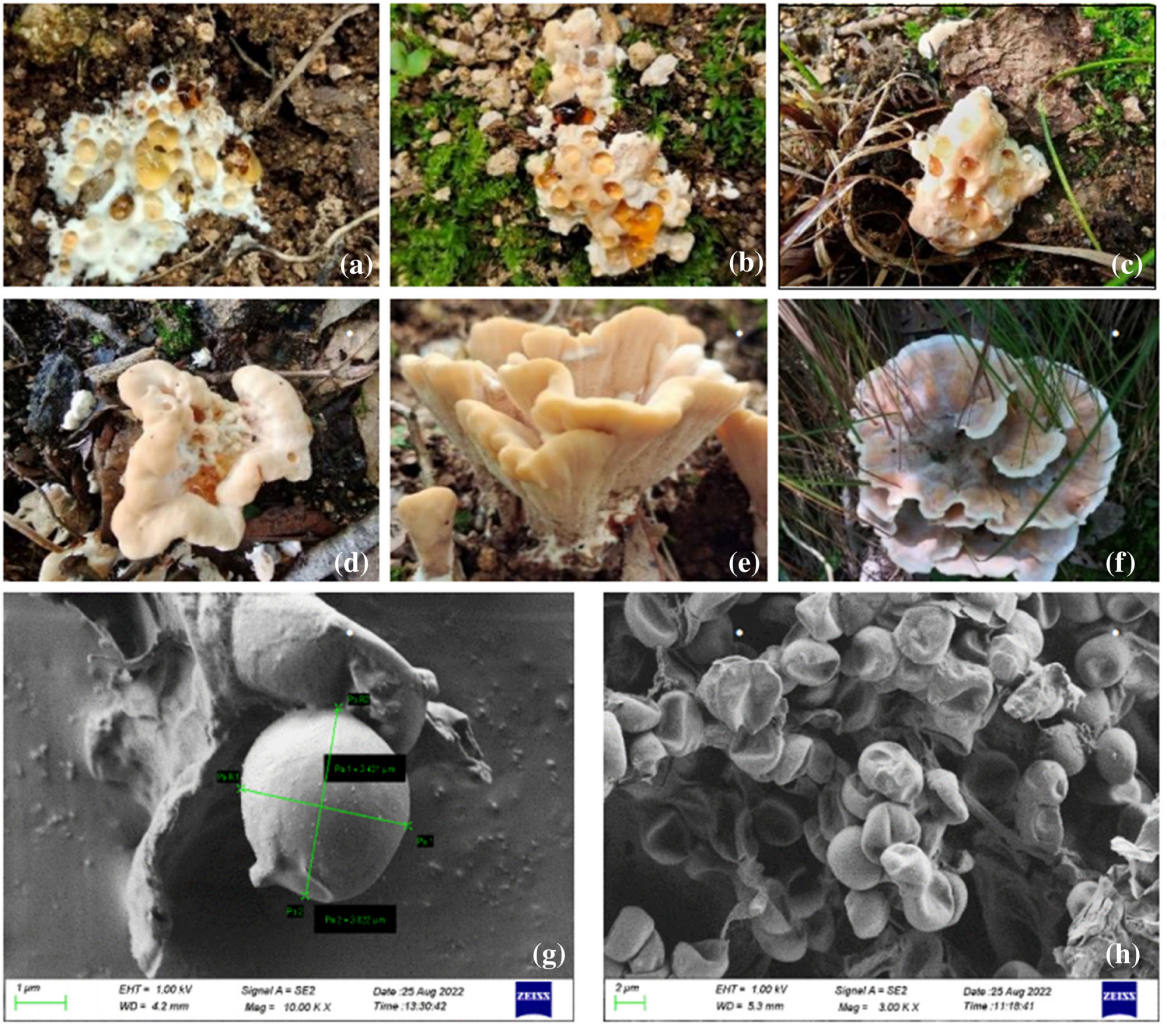
Morphological characteristics and spore morphology of *Abortiporus baotianmanensis*. Note: (a), (b), (c), (d), (e), and (f) are macromorphic habitat images of *Abortiporus baotianmanensis* in different growth periods; (g) and (h) are the basidiospore characteristics of *Abortiporus baotianmanensis* residue under an electron microscope.

During the macrofungi survey in the Baotianman Biosphere Reserve, a species of polyporaceae was collected, and the species could not be determined by morphological comparison. After the tissue isolation was made in the laboratory, the strains were obtained, and their DNA was extracted. After the molecular identification and comparison, it was found that by searching with BLAST on the NCBI website, the support rate was lower than 97.5%, and the morphology and habitat were quite different from those of *Abortiporus biennis*. It might be a new species of *Abortiporus*. A phylogenetic tree was then constructed for further verification [28]. As shown in Figure 2, the new species of *Abortiporus baotianmanensis* (M41AA30) was clustered into a single branch, which confirmed the above conclusion.

**Figure 3:**
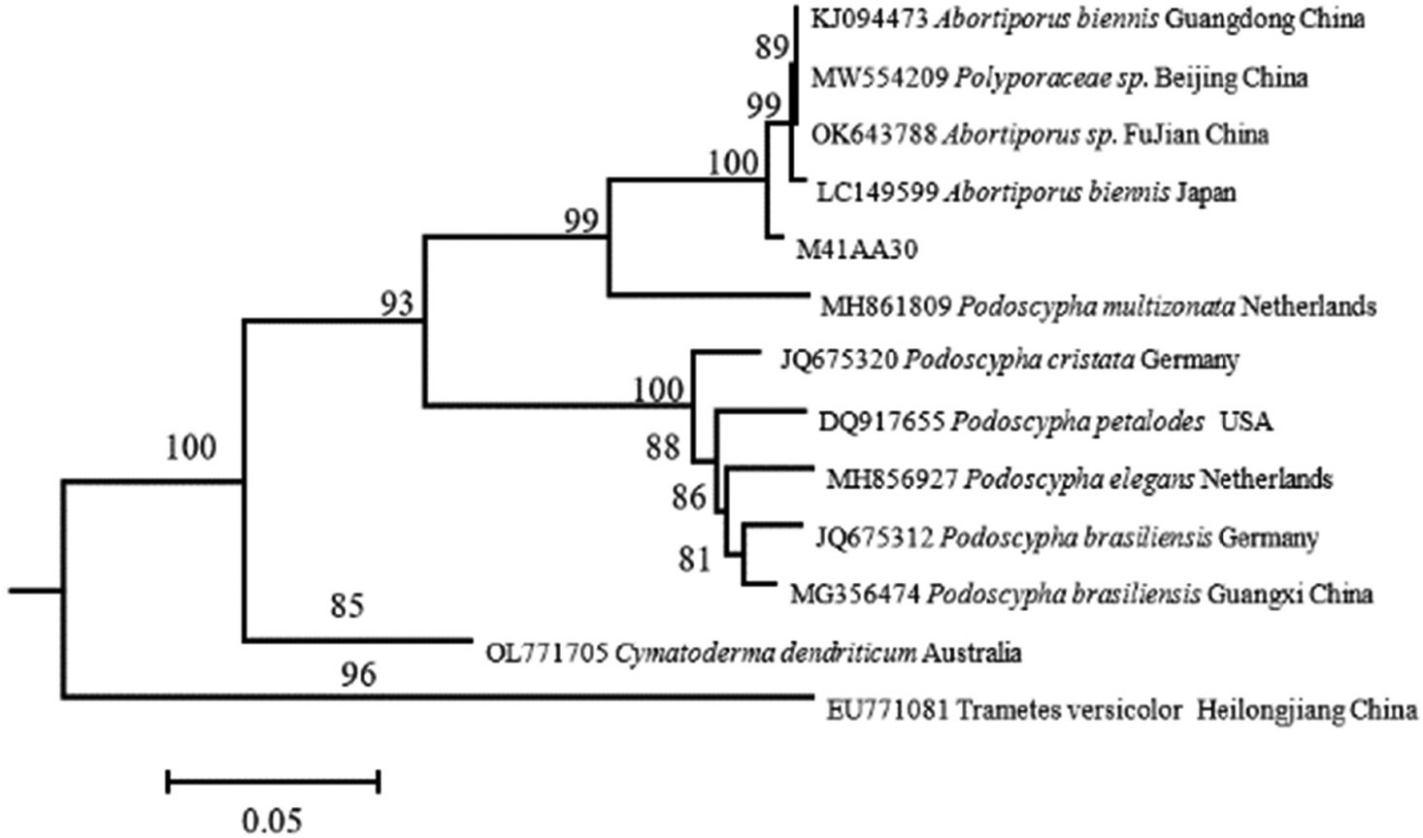
Phylogenetic tree based on ITS sequences.

A phylogenetic tree was constructed, the information of the target macrofungi and related genera and species was queried in the fungal index database, and the information of species with high similarity was found on the BlAST in the NCBI website. All sequences were summarized in a.txt file which was changed to a.fasta file. The changed file was opened with MEGA 6.0, all sequences were aligned, and the bases that were not aligned at both ends were cut manually and saved as a.meg file. It was reopened with MEGA 6.0, and a phylogenetic tree was built for analysis with the Neighbor-joining method ([NJ] for selected sequences (Table 6 and Figure 3).

**Table 6:** Sequence table for phylogenetic analysis of *Abortiporus baotianmanensis* (new species)

No.	Species	Specimen No.	Genbank No. ITS	Origin
1	—	M41AA30	—	Henan China
2	*Abortiporus biennis*		LC149599	Japan
3	*Abortiporus* sp.		OK643788	Fujian China
4	*Abortiporus biennis*		KJ094473	Guangdong China
5	*Polyporaceae* sp.		MW554209	Beijing China
6	*Podoscypha brasiliensis*		JQ675312	Germany
7	*Podoscypha brasiliensis*		MG356474	Guangxi China
8	*Podoscypha gillesii*		MG356710	Guangxi China
9	*Cymatoderma dendriticum*		OL771705	Australia
10	*Podoscypha cristata*		JQ675320	Germany
11	*Podoscypha elegans*		MH856927	Netherlands
12	*Trametes versicolor*		EU771081	Heilongjiang China
13	*Podoscypha petalodes*		DQ917655	USA
14	*Podoscypha multizonata*		MH861809	Netherlands

The morphology of *Abortiporus baotianmanensis* was preliminarily described as follows (Figure 2): The fruiting bodies are annuals, polyporaceae-like, medium to large; imbricate-like overlapping, without or with short stipe when growing from the side of the base, rosette-like overlapping with short stipe when growing from the top of the base. The cap is fan-shaped, extending up to 15 cm in length, 24 cm in width, and 11 mm in thickness, white, cream, to light orange-brown in the young period, and can secrete yellow-brown to ruby-red droplets, with thick, blunt, and wavy edges; wine reddish-brown in the ripe period, fading to white, cream-colored margins, with distinct concentric bands, and thin and sharp wavy to valved margins. The context has a mushroom odor, tasteless and heterogeneous; the upper layer is cream-colored to light orange-brown, not discolored after damage, spongy; the lower layer is a light wood color, not discolored after damage, suberous. The fruiting layer is irregular, polygonal tubular, labyrinth-like to pleated, obvious in the young period, up to 4 mm in length, gradually becomes smooth in the ripe period, white to creamy, and turns dark reddish-brown when being touched. The basidiospores are broadly oval and smooth, with a size of 4.5–6.0 × 3.0–4.5 μm.

Since the newly identified species (*Abortiporus baotianmanensis*) of podoscyphaceae belongs to the genus *Abortiporus*, which has great potential for therapeutic use, it offers very helpful insights for the future. For instance, *Abortiporus biennis* (Bull.) Singer belongs to the phylum Basidiomycotina, Agaricomycetes, Aphyllophorales, Polyporaceae, and *Abortiporus*. It is born in broad-leaved trees such as oak, maple, and apple, or on the ground buried with decayed wood. It is a kind of white rot fungus, but also a kind of medicinal fungus, that has the effect of tumor inhibition [29]. It is a worldwide species [30,31,32,33,34]. Because of its rich enzyme system, it has been reported to be used in the study of solid-state fermented straw [35]. There have also been in-depth studies on the changes in cell structure and chemical substances under heavy metal stress [36,37,38]. Environmental microbial experiments were also conducted, such as paraquat dichloride and C14 lignocellulose [39,40,41,42]. Because of these uses, *Abortiporus baotianmanensis* can be studied more thoroughly for its potential therapeutic properties.

## 4 Conclusion

The Baotianman Biosphere Reserve offers macrofungi abundant resources. Its unique ecological habitat is ideal for scientific research. However, the Reserve lacks systematic macrofungal research. Hence, this study examined macrofungi’s floristic composition, species diversity, and interaction with plant communities. As a result, 832 specimens were collected using sampling and random inspection techniques, 351 species of macrofungi were identified, and a new species of suspected macrofungi was discovered. The species-level diversity index analysis of macrofungi among the four vegetation types in the reserve showed significant differences in macrofungal diversity between communities, suggesting that vegetation types have a greater influence on macrofungi. A total of 196 edible fungi, 121 medicinal fungi, 52 poisonous fungi, and 37 macrofungi with unclear economic value were assessed for macrofungal resources. Due to the high concentration of economically useful macrofungi, the reserve has great potential for growth and use. Based on traditional taxonomy, molecular assistance, and phylogenetic analysis, the new macrofungi species *Abortiporus baotianmanensis* was found to be an unreported podoscyphaceae in the genus *Abortiporus*. This article addresses a knowledge gap in the research on macrofungi found in the Baotianman Biosphere Reserve. The identification of a newly discovered species demonstrates the reserve’s abundant biodiversity. More fungi resources will be found, and a tentative macrofungi conservation system in the Baotianman World Biosphere Reserve will provide a theoretical basis for future growth and application.
